# Polariton polarization rectifier

**DOI:** 10.1038/s41377-019-0189-z

**Published:** 2019-08-28

**Authors:** Evgeny S. Sedov, Yuri G. Rubo, Alexey V. Kavokin

**Affiliations:** 1 Westlake University, 18 Shilongshan Road, Hangzhou, 310024 Zhejiang Province China; 2 Institute of Natural Sciences, Westlake Institute for Advanced Study, 18 Shilongshan Road, Hangzhou, 310024 Zhejiang Province China; 3 Vladimir State University, Gorky str. 87, Vladimir, 600000 Russia; 4 Instituto de Energías Renovables, Universidad Nacional Autónoma de México, 62580 Temixco, MOR Mexico; 5 Spin Optics Laboratory, Saint Petersburg State University, 1 Ulianovskaya, St. Petersburg, 198504 Russia

**Keywords:** Optoelectronic devices and components, Polaritons

## Abstract

We propose a novel photonic device, the polariton polarization rectifier, intended to transform polariton pulses with arbitrary polarization into linearly polarized pulses with controllable orientation of the polarization plane. It is based on the interplay between the orbital motion of the polariton wave packet and the dynamics of the polariton pseudospin governed by the spatially dependent effective magnetic field. The latter is controlled by the TE-TM splitting in a harmonic trap. We show that the unpolarized polariton pulse acquires linear polarization in the course of propagation in a harmonic trap. This gives the considered structure an extra function as a linear polarizer of polariton pulses.

## Controlling the polariton

Numerical simulations show how a light-based device can manipulate the oscillatory planes of polaritons, offering more control over these quasiparticles and paving the way towards faster, less energy-consuming electronics. Evgeny Sedov of Westlake University, China, and colleagues demonstrated that a polariton pulse of any polarization can be split into two pulses of unequal intensity that are at right angles to each other. Their intensities can be controlled, to the point that one pulse could be completely suppressed. Polaritons form when light photons confined in one direction interact with matter excitations in a thin semiconductor. These low mass, light-like quasiparticles have huge potential for applications in computing and signal processing. This study adds to the body of research aimed at studying their unique properties and learning how to control them.

## Introduction

The concept of polariton devices is based on manipulation by macroscopic coherent states of quasiparticles exciton-polaritons appearing under the strong coupling of excitons in semiconductor crystals with quantized light in optical microcavities^1^. Their properties combine the mobility and flexibility of photons^2^ and the controllability of excitons^3^, which allows polariton devices to be competitive with traditional photonic and electronic devices for light control and signal processing. Polaritons inherit a spin degree of freedom from both their constituents^4^, which gives access to the polarization properties of the emitted light. Controlling the polariton spin relaxation processes is an effective way to tailor light polarization. The polariton pseudospin (Stokes vector) is in the heart of various fundamental effects, including the optical spin Hall effect^5–7^, polarization bistability^8^ and multistability^9,10^, spin currents of exciton polaritons^6,11^, etc. The most considerable mechanism of the polariton spin relaxation is the precession of polariton pseudospins induced by the splitting of the transverse electric and transverse magnetic photonic microcavity modes (TE-TM splitting)^12^ along with the long-range electron and hole exchange interaction^13^. The effect of the longitudinal-transverse splitting on the pseudospin behaviour can be described in terms of the effective quasimomentum-dependent in-plane magnetic field **Ω**(**k**)^14^. Methods for controlling the spin degree of freedom are used in a set of devices referred to as spinoptronic devices^3^ for manipulation by light polarization, including polariton polarization switches^15^, polarization filters of polariton flows^16^, polarization-controlled optical gates^17^, and the Berry-phase interferometer^18^.

Among established methods of controlling the polariton spin degree of freedom is the creation of an external confining potential. Flat one-dimensional polariton waveguides have been widely studied from the perspective of the creation and propagation of spin-split polariton condensates^19^ and solitonic polariton pulses^20^ as well as of accompanying effects, including backward Cherenkov radiation^21^, and the formation of polarization domains^22^. Recently, ring-shaped geometry was recognized in the study of polariton polarization behaviour^18,23–25^. In this geometry, the in-plane effective magnetic field acting upon a polariton pseudospin rotates as the polariton moves along the circle around the symmetry axis of the ring-shaped confining potential characterized by the frequency *ω*_tr_, which enriches the polarization dynamics with the complementary oscillation degree in addition to the Larmor precession Ω(*k*). Typically, the consideration of the polariton dynamics as well as the polarization dynamics in spin-split systems is performed within the adiabatic limit, assuming that the spectral width of the polariton state Δ*ω* does not exceed the characteristic frequency scales of the system, Δ*ω* < *ω*_tr_, Ω(*k*), as well as the separation of the energy levels, which is significant in the narrow confining potential. Furthermore, the limit where the Larmor frequency is considered the largest frequency in the system, $\Omega (k) \gg \Delta \omega ,\,\omega _{{\text{tr}}}$, is used to separate different oscillation scales^18^. In this approach, the internal behaviour of the polariton pulse as well as the interaction of polariton modes is adiabatically eliminated from consideration, and its dynamics is described by the effective 1D Hamiltonian for a classical spinning particle. The situation dramatically changes when the internal pulse behaviour is not completely suppressed and polaritons are being filtered by their wave vectors such that their distribution changes in the radial direction. This scenario may be realized in broad confining potentials, including the harmonic potential.

In this paper, we consider the polarization dynamics of polariton pulses in a harmonic potential beyond the adiabatic limit. We demonstrate the splitting in real space of an injected polariton pulse of any given polarization into two pulses of unequal intensity with orthogonal linear polarizations. The intensities of the resulting pulses can be manipulated, including complete suppression of one of them. Based on the obtained peculiar polarization behaviour, we propose a novel spinoptronic device, the polariton polarization rectifier, which transforms the arbitrary polarization of the optical pulse to a linear polarization with controlled orientation of the polarization plane. We also show that the proposed device assigns linear polarization to a polariton pulse excited by an unpolarized resonant probe.

## Results

### The concept of the device

**Fig. 1:**
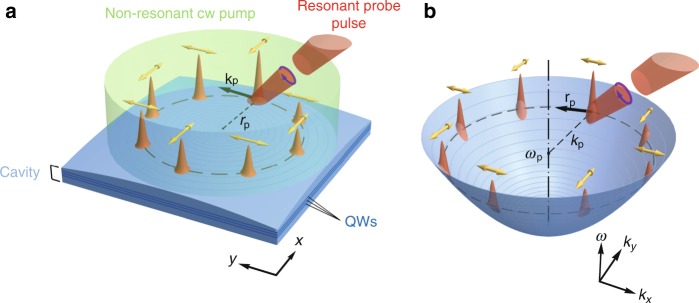
Schematic of the possible experimental configuration of the polariton polarization rectifier. **a** Sketch of the possible experimental configuration. The polariton condensate is excited by a resonant probe pulse (red split cone) at a distance *r*_p_ from the centre of the harmonic trap with the wave vector *k*_p_ and energy $\hbar \omega _{\text{p}}$, allowing the polariton pulse to propagate along a circular trajectory (dashed circle). The subthreshold non-resonant cw pump (pale green) is used to increase the pulse lifetime. **b** Potential landscape across the structure in the reciprocal space. The yellow arrows in both panels indicate the orientation of the linear polarization plane of the propagating polariton pulse at different positions along the propagation trajectory. The polarization of the probe pulse can be chosen arbitrarily. In panels (**a**) and (**b**), the circular polarization (blue arrow) is shown as an example. The blue concentric circles in both panels are given as a guide for the eye to indicate the harmonic trap

The geometry of the device setup is shown schematically in Fig. 1a. It consists of an optical microcavity with an embedded set of quantum wells operating in a strong-coupling regime. Arising exciton-polaritons are confined in an in-plane harmonic potential, which can be created either for excitons via application of a local stress to the sample^26,27^ or for photons by creating mesas^28,29^ with thicknesses varying along their radius. The latter option is illustrated in Fig. 1a. In Fig. 1b, the potential landscape across the structure is schematically shown in the reciprocal space. Polaritonic systems also allow the use of optical traps^30^ created by the non-resonant optical pump beam of a given shape. Polaritons are injected by a resonant pulsed probe of energy $\hbar \omega _{\text{p}}$ at a distance *r*_p_ from the centre of the trap tangential to its surface with the quasimomentum *k*_p_. To increase the polariton pulse lifetime, we introduce the subthreshold spatially homogeneous non-resonant cw optical pumping of excitons.

In the conservative limit, the system is described by the effective Hamiltonian

1 $$\hat H_0 = \frac{{\hbar ^2{\hat{\mathbf k}}^2}}{{2m^ \ast }} + V\left( {\mathbf{r}} \right) + \hbar {\hat{\boldsymbol{\Omega} }} \cdot {\hat{\mathbf S}}$$

where $m^ \ast = 2m_{\text{l}}m_{\text{t}}/\left( {m_{\text{l}} + m_{\text{t}}} \right)$, with *m*_l_ and *m*_t_ being the effective masses of longitudinal (or transverse-magnetic, TM) and transverse (or transverse-electric, TE) polariton modes, respectively; ${\mathbf r} = \left( {x,y} \right)$ and ${\hat{\mathbf k}} = \left( {\hat k_x,\hat k_y} \right)$ are the polariton position and quasimometum operators, respectively. $V\left( {\mathbf{r}} \right) = m^ \ast \omega _{{\text{tr}}}^2(x^2 + y^2)/2$ is the harmonic potential characterized by the frequency *ω*_tr_. The polariton quantum state is described by the spinor $\left. {|{\mathrm{\Psi }}({\mathbf{r}})} \right\rangle = [{\mathrm{\Psi }}_ + ({\mathbf{r}}),{\mathrm{\Psi }}_ - ({\mathbf{r}})]^{\text{T}}$. We introduce the three-component spin operator ${\hat{\mathbf S}} = \frac{1}{2}{\hat{\boldsymbol{\sigma} }}$, where ${\hat{\boldsymbol{ \sigma} }} = ({\hat{\mathrm \sigma }}_x,{\hat{\mathrm \sigma }}_y,{\hat{\mathrm \sigma }}_z)$ is the vector of the Pauli operators. The TE-TM splitting gives rise to the directionally dependent effective magnetic field ${\hat{\boldsymbol{\Omega} }} = \left[ {\Delta _{{\text{LT}}}\left( {\hat k_x^2 - \hat k_y^2} \right),\,2\Delta _{{\text{LT}}}\hat k_x\hat k_y,\,0} \right]$ in the microcavity plane, which causes precession of the polariton pseudospin with the effective Larmor frequency $\Omega (k)$. $\Delta _{{\text{LT}}} = (\hbar /2)\left( {m_{\text{l}}^{ - 1} - m_{\text{t}}^{ - 1}} \right)$ is the TE-TM splitting constant.

In the spin-degenerate case (Δ_LT_ = 0), the Hamiltonian (1) represents the 2D quantum harmonic oscillator and is integrable. According to the correspondence principle^31^, at high energies, the quantum treatment of a wave function behaviour merges with the classical one of a single particle. The dynamics of a polariton pulse is well described by the classical equations for the trajectory of its centre of mass: $d_{\text{t}}\left\langle {\mathbf{r}} \right\rangle \left( t \right) = \left\langle {\mathbf{k}} \right\rangle \left( t \right)/m^ \ast$, $d_{\text{t}}\left\langle {\mathbf{k}} \right\rangle \left( t \right) = - m^ \ast \omega _{{\text{tr}}}^2\left\langle {\mathbf{r}} \right\rangle \left( t \right)$. The pulse propagates along the closed elliptical orbit, which degenerates to a circular orbit when the initial conditions $\left\langle {\mathbf{r}} \right\rangle \left( 0 \right) = {\mathbf{r}}_0$, and $\left\langle {\mathbf{k}} \right\rangle \left( 0 \right) = {\mathbf{k}}_0$, obey the condition of equality of the kinetic and potential energies, $\hbar ^2k_0^2/2m^ \ast = V(r_0)$.

**Fig. 2:**
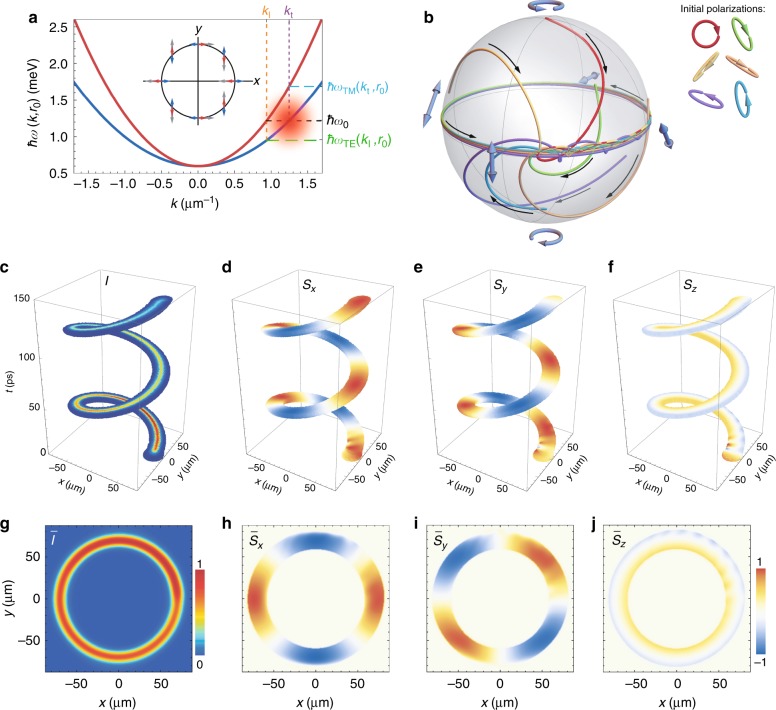
Polarization behaviour of a polariton pulse excited resonantly to the TE dispersion branch. **a** Dispersion of two spin-split polariton modes $\hbar \omega _{{\text{TE}},{\text{TM}}}(k,r_0)$ at position *r*_0_ = 70 μm. The origin zero energy corresponds to $\hbar \omega (0,0)$. The red blurry spot indicates the position and schematic spectral distribution of the probe pulse. The probe pulse of 5-ps duration is resonant to the TE polariton mode, i.e., *k*_p_ = *k*_t_ and *ω*_p_ = *ω*_0_ at *r*_0_. The inset schematically shows the orientation in real space of the effective magnetic field **Ω**(**k**) (grey arrows) and the pseudospin of polaritons belonging to the TE (blue arrows) and TM (red arrows) branches. **b** Trajectories on the Poincaré sphere of the Stokes vector characterizing polarization of the polariton pulse for different polarizations of the probe pulse (indicated in the panel). The black arrows indicate the direction with respect to the evolution in time. **c** Evolution of the intensity and **d**–**f** polarization components of the polariton pulse. **g** Time-integrated spatial distribution of the normalized intensity and **h**–**j** polarization components of the polariton pulse. The polarization of the probe pulse is taken as right circular for (**c**)–(**j**)

In the presence of TE-TM splitting, the Hamiltonian (1) is not integrable, and the vast majority of semiclassical trajectories are not closed. There remain, however, two important types of exact solutions, that is, those describing the polariton motion along circular orbits with always either transverse (TE) or longitudinal (TM) spin. The energies of these circular modes are defined by $\hbar ^2k_{{\text{l}},{\text{t}}}^2/2m_{\text{l},{\text t}} = V(r_0)$, resulting in different momenta for the same radius of the orbit *r*_0_. In Fig. 2a, we show the energies of two polariton modes $\hbar \omega _{{\text{TM}},{\text{TE}}}\left( {k,r_0} \right) = V(r_0) + \hbar ^2k^2/2m_{{\text l},{\text t}}$ in the sample at position *r*_0_ = 70 μm. (Values of other parameters are given in the Methods section.) A circular trajectory of radius *r*_0_ is inherent to polaritons belonging to the TM (upper red curve) and the TE (lower blue curve) branches excited with the energy $\hbar \omega _0 = \hbar \omega _{{\text{TM}}}\left( {k_{\text l},r_0} \right) = \hbar \omega _{{\text{TE}}}\left( {k_{\text t},r_0} \right)$ and the momentum *k*_l_ and *k*_t_, respectively.

A Gaussian resonant pulse excites different eigenmodes in the parabolic trap. For sufficiently strong TE-TM splitting, most of them propagate irregularly in the trap and destructively interfere with each other. In contrast, the longitudinal and transverse wave packets are able to propagate long azimuthal distances without substantial changes in their shapes. Their pseudospins follow the direction of the effective magnetic field and are co- and counterdirected to it for TM and TE modes, respectively. This implies that the electric field oscillates in the azimuthal direction for the longitudinal (TM) mode and oscillates in the radial direction for the transverse (TE) mode. The orientation of the corresponding polariton pseudospins during evolution is schematically shown in the inset of Fig. 2a. A resonantly excited polariton pulse of finite width and duration possesses the energy and quasimomentum spectrum, overlapping with the eigenstates of the system. This implies that in the course of evolution, regardless of the initial polarization, the polariton pulse tends to occupy the longitudinal and transverse eigenstates and assigns linear polarization characteristics to them.

### Polarization dynamics of polariton pulses

To reveal the polarization dynamics of polariton pulses in the proposed structure, we perform a set of numerical simulations based on the generalized Pauli equation for the spinor $\left. |\mathrm{\Psi} \right\rangle = \left[ \mathrm{\Psi}_{+} (t,\mathbf{r}),\mathrm{\Psi}_ {-} (t,\mathbf{r}) \right]^{\text{T}}$, where $\mathrm{\Psi}_ {\pm} (t,\mathbf{r})$ are the wave functions of the right- and left-circularly polarized polariton components; see details of the model in the Methods section. In our first numerical experiment, we consider the behaviour of the polariton pulse excited by the Gaussian probe pulse of duration *w*_T_ = 5 ps resonant to the TE polariton branch, i.e., characterized by the central frequency *ω*_p_ = *ω*_0_ and the central wave vector **k**_p_ = (0, *k*_t_). The excitation spectrum in comparison with the polariton dispersion is schematically shown in Fig. 2a. The pulse is excited at position **r**_0_ = (*r*_0_, 0). We take the pump distance as *r*_0_ = 70 μm. For the kinetic energy of polaritons to match the potential energy and the polariton pulse to follow a closed circular trajectory, the corresponding quasimomentum *k*_t_ should be as large as $k_{\text{t}} \simeq 1.245\,{{\upmu}}{\text{m}}^{ - 1}$. A sufficiently large quasimomentum is essential to reduce the effect of the *zitterbewegung*, i.e., the trembling of the trajectory of polaritons in real space due to the influence of the spin degree of freedom. As we have shown in ref. ^32^, the *zitterbewegung* is less pronounced at *k* *>* 1 µm since its amplitude is inversely proportional to the quasimomentum.

Figure 2c shows the evolution in time of the spatial intensity distribution $I\left( {t,{\mathbf{r}}} \right) = {\mathrm{\Psi }}^\dagger (t,{\mathbf{r}}){\mathrm{\Psi }}(t,{\mathbf{r}})$ of the polariton pulse excited by the right-circularly polarized probe pulse, $\left. {|f} \right\rangle = \left[ {1,0} \right]^{\text{T}}$. In Fig. 2g, the ring-shaped distribution of intensity of the polariton pulse, integrated over the time of observation, $\bar I({\mathbf{r}}) = {\int} {I(t,{\mathbf{r}}){\text d}t}$, is presented. The ring thickens near the polariton injection spot around *r*_0_. The evolution of the spatial distribution of the polarization components $S_j\left( {t,{\mathbf{r}}} \right) = {\mathrm{\Psi }}^\dagger \left( {t,{\mathbf{r}}} \right)\hat S_j{\mathrm{\Psi }}\left( {t,{\mathbf{r}}} \right)/I\left( {t,{\mathbf{r}}} \right)$ $(j = x,y,z)$ of the polariton pulse is shown in Fig. 2d–f, accompanied by the integrated in time distribution of the polarization, $\bar S_j\left( {\mathbf{r}} \right) = {\int} {\Psi ^\dagger (t,{\mathbf{r}})\hat S_j{\mathrm{\Psi }}(t,{\mathbf{r}}){\text d}t/\bar I(t,{\mathbf{r}})}$, in Fig. 2h–j. Summarizing the main peculiarities of the figures, we can claim that regardless of the initial circular polarization, the latter is barely presented in the resulting polarization map. The polariton pulse possesses linear polarization, and the polarization plane twice rotates around the centre of the harmonic trap, adjusting the rotation of the effective magnetic field ${\hat{\boldsymbol{\Omega} }}({\mathbf{k}})$. At the beginning of the evolution, the polarization components experience fast oscillations with a frequency $\Delta _{{\text{LT}}}k_0^2$, which result in the appearance of the periodic ripples most visible at the periphery of the polarization patterns in the upper-right quadrant in the colour maps (Fig. 2h−j). The oscillations, however, possess a transient character and disappear at approximately a quarter of the first pass around the trap.

The more important result is that under the considered excitation conditions, the polariton pulse acquires linear polarization regardless of the initial polarization of the probe. In Fig. 2b, we show trajectories of the normalized Stokes vector ${\mathbf{S}}\left( t \right) = \left\langle {{\mathrm{\Psi }}|{\hat{\mathbf S}}|{\mathrm{\Psi }}} \right\rangle /\left\langle {{\mathrm{\Psi }}|{\mathrm{\Psi }}} \right\rangle$ on the Poincaré sphere for various initial conditions. In all numerical experiments presented, the degree of the circular polarization in the polariton pulse after one period of rotation around the harmonic trap does not exceed half a percent.

In the considered excitation circumstances, the width of the spatial spectrum of the probe pulse $2\pi /w \approx 0.77\,{\upmu} \text{m}^{ - 1}$ considerably exceeds the splitting of the TE and TM branches, $k_{\text{t}} - k_{\text{l}} \approx 0.31\,{\upmu} {\text{m}}^{ - 1}$. Although the pulse spectrum is centred with respect to the TE mode, it nevertheless partially overlaps with the TM mode. However, despite this overlap, the TM mode is not presented in the pulse dynamics in Fig. 2.

**Fig. 3:**
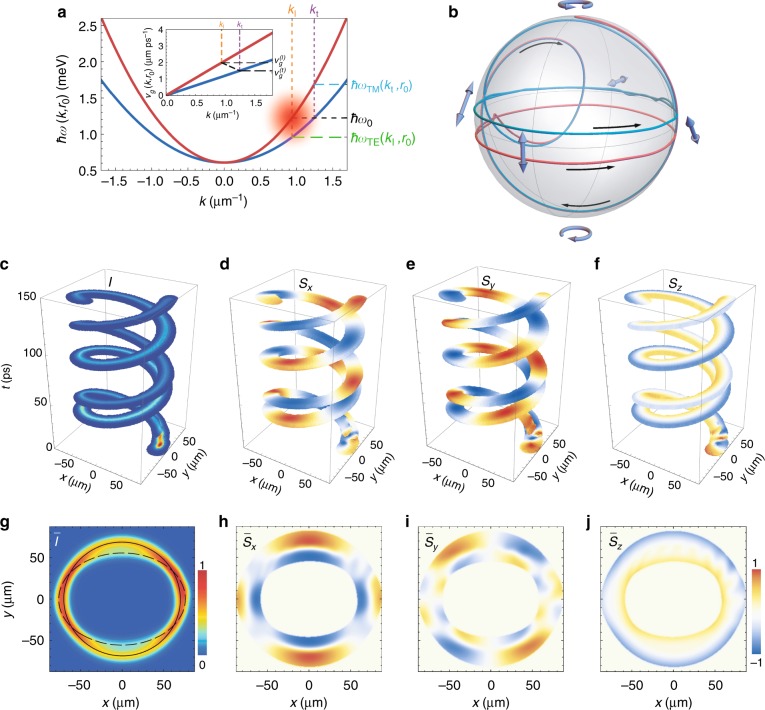
Polarization behaviour of a polariton pulse excited resonantly to the TM dispersion branch. **a** Dispersion of two spin-split polariton modes $\hbar \omega _{{\text{TE}},{\text{TM}}}(k,r_0)$ at position *r*_0_ = 70 μm in comparison with the spectrum of the probe pulse. The probe pulse of 5-ps duration is resonant to the TM polariton mode, i.e., *k*_p_ = *k*_l_ and *ω*_p_ = *ω*_0_ at *r*_0_. The inset shows group velocities $v_{\text g}^{{\text{TE}},{\text{TM}}}(k)$ of polaritons belonging to the TE and TM branches as functions of *k*. **b** Trajectories on the Poincaré sphere of the Stokes vector characterizing polarization of the two spatio-temporal components of the split polariton pulse. The black arrows indicate the direction with respect to the evolution in time. **c** Evolution of the intensity and **d**–**f** polarization of the two components of the polariton pulse. **g** Time-integrated spatial distribution of the normalized intensity and **h**–**j** polarization components of the polariton pulse. The polarization of the probe pulse is taken as right circular for (**c**)–(**j**). The dashed and solid closed black curves in panel (**g**) indicate the trajectories of the TE and TM components of the polariton pulse, respectively

In the next numerical experiment, we keep the probe energy as $\hbar \omega _0$ but take the probe wave number as *k*_p_ = *k*_l_ such that the probe is now resonant to the TM branch; see the comparison of the excitation spectrum with the polariton dispersion in Fig. 3a. The initial polarization is again taken as right circular. The polariton pulse dynamics and the dynamics of the polarization components are presented in Fig. 3c–f, accompanied by the time-integrated intensity and polarization degree in Fig. 3g–j. In contrast to the above-considered case, the initial single wave packet, over the course of evolution, splits into two components of orthogonal polarizations; see the two spiral trajectories in Fig. 3c–f. The more intensive wave packet component belongs to the resonantly excited TM branch, while the less intensive component belongs to the TE branch.

The two components of the polariton pulse demonstrate different behaviours. The inset in Fig. 3a shows the group velocity of polaritons belonging to the TE and TM branches, $v_{\mathrm g}^{\left( {{\text{TE}},{\text{TM}}} \right)}\left( {k,r_0} \right) = \partial _{\text{k}}\omega _{{\text{TE}},{\text{TM}}}(k,r_0)$. It is clearly seen that for the same probe energy $\hbar \omega _{\text{p}} = \hbar \omega _0$, the group velocity of the TM component of the pulse, $v_{\text g}^{\left( {\text l} \right)} = v_{\text g}^{\left( {{\text{TM}}} \right)}\left( {k_{\text l},r_0} \right)$, is larger than that of the TE component, $v_{\text g}^{\left( {\text t} \right)} = v_{\text g}^{\left( {{\text{TE}}} \right)}\left( {k_{\text t},r_0} \right)$: $v_{\text g}^{\left( {\text l} \right)} \,{>}\, v_{\text g}^{\left( {\text t} \right)}$.

The considered case is also remarkable because the orthogonal linear polarizations of the polariton wave packet are separated in the radial direction: the $\bar S_x \,{>}\, 0$ component is concentrated in the outer region, while the $\bar S_x \,<\, 0$ component is concentrated in the inner region of the highlighted area of the colour map in Fig. 3h. The dashed and solid closed black curves in Fig. 3g show the trajectories of the TE and TM components, respectively, of the polariton pulse described by the parametric dependence *Y*_*j*_(*X*_*j*_) for $X_j\left( t \right) = \mathop {\int }\nolimits_{A_j}^{} I(t,{\mathbf{r}})x{\text d}{\mathbf{r}}/\hskip -4pt\mathop {\int }\nolimits_{A_j}^{} I(t,{\mathbf{r}}){\text d}{\mathbf{r}}$ and $Y_j\left( t \right) = \mathop {\int }\nolimits_{A_j}^{} I(t,{\mathbf{r}})y{\text d}{\mathbf{r}}/\hskip -4pt\mathop {\int }\nolimits_{A_j}^{} I(t,{\mathbf{r}}){\text d}{\mathbf{r}}$, where *j* *=* TE, TM; *A*_TE_ and *A*_TM_ indicate the areas on the cavity plane occupied by the corresponding components of the polariton pulse. The trajectory of the TM pulse component is nearly circular, as the excitation is symmetric relative to the probe quasimomentum *k*_p_ = *k*_l_, and the kinetic energy matches the potential energy of the wave packet. In contrast, the TE pulse component propagates along the elliptical trajectory, with the major axis oriented along the *x* axis. This is connected with the fact that the TE component of the pulse that is distant from the excitation quasimomentum by *k*_t_ − *k*_p_ is excited with the quasimomenta *k* on the periphery of the excitation pulse far from the central quasimomentum, *k*_p_ < *k*. Hence, the central wave vector of the TE component of the pulse is shifted to lower *k* relative to *k*_t_ for which the kinetic and potential energy matching condition is not satisfied. This results in the elliptical shape of the trajectory. In the considered excitation scheme, the orientation of the minor axis of the elliptical trajectory of the TE component of the polariton pulse coincides with the *y* axis. Hence, the polarization of the TE pulse component near *x* = 0 (one can briefly characterize it as $\bar S_x \,<\, 0$) is concentrated inside the coloured region of the colour maps in Fig. 3h–j.

Figure 3b shows the trajectories of the normalized Stokes vector **S**(*t*) on the Poincaré sphere of the two components of the pulse. In both components, the circular polarization is presented by a small quantity of less than 5%. The residual circular polarization is right circular for the TE-branch pulse and left circular for the TM-branch pulse.

**Fig. 4:**
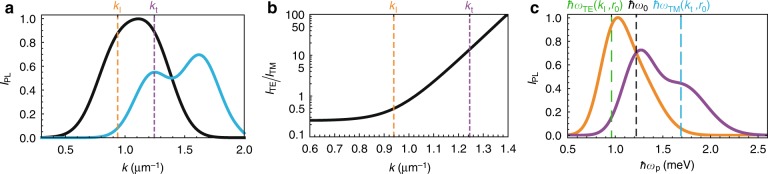
The intensity of polariton pulses depending on the excitation conditions. **a** The dependence of the total time-integrated intensity of the polariton pulse on the probe wave number *k*_p_ for two probe energies, $\hbar \omega _{\text{p}} = \hbar \omega _0$ (black curve) and $\hbar \omega _{\text{p}} = \hbar \omega _{{\text{TE}}}(k_{\text{l}},r_0)$ (blue curve). **b** The ratio of the time-integrated intensities of two spatial components, *I*_TE_ and *I*_TM_, of the polariton wave packet as a function of the probe wave number at the probe energy $\hbar \omega _{\text{p}} = \hbar \omega _0$. **c** The total time-integrated intensity of the polariton wave packet as a function of the probe energy $\hbar \omega _{\text{p}}$ at two resonant quasimonenta, *k*_l_ (orange curve) and *k*_t_ (purple curve)

To further investigate the resonant character of the polarized polariton pulse excitation, in Fig. 4a, we plot the dependence of the total intensity of two pulse components integrated in time on the probe wave number *k*_p_ for two probe energies, $\hbar \omega _{\text{p}} = \hbar \omega _0$ (black curve) and $\hbar \omega _{{\text{TM}}}(k_{\text{t}},r_0)$ (blue curve). The latter energy corresponds to the resonant energy on the TM branch at *k*_t_. The ratio of the time-integrated intensities of two spatial components of the polariton wave packet as a function of the probe wave number at the probe energy $\hbar \omega _{\text{p}} = \hbar \omega _0$ is presented in Fig. 4b. Figure 4a shows that the total intensity of the polariton wave packet is nearly identical at two resonant quasimomenta, *k*_l_ and *k*_t_, corresponding to the cases considered in Figs. 2 and 3. However, the key difference between the cases, that is, the very significant domination of one component of the polariton wave packet over the other at *k*_p_ = *k*_t_, is highlighted in Fig. 4b. The intensity of the dominant component at *k*_t_ exceeds that of the other component by more than one and half orders, while at *k*_l_, the superiority is less than three times. Figure 4c shows the total time-integrated intensity of the polariton wave packet as a function of the probe energy $\hbar \omega _{\text{p}}$ at two resonant quasimonenta, *k*_l_ (orange curve) and *k*_t_ (purple curve). In both dependencies, the intensity reaches its maximum value near the resonance with the TE-polarized branch: close to *ω*_t_ at *k*_p_ = *k*_l_ and close to *ω*_0_ at *k*_p_ = *k*_t_; cf. with the dispersion in Fig. 3a.

**Fig. 5:**
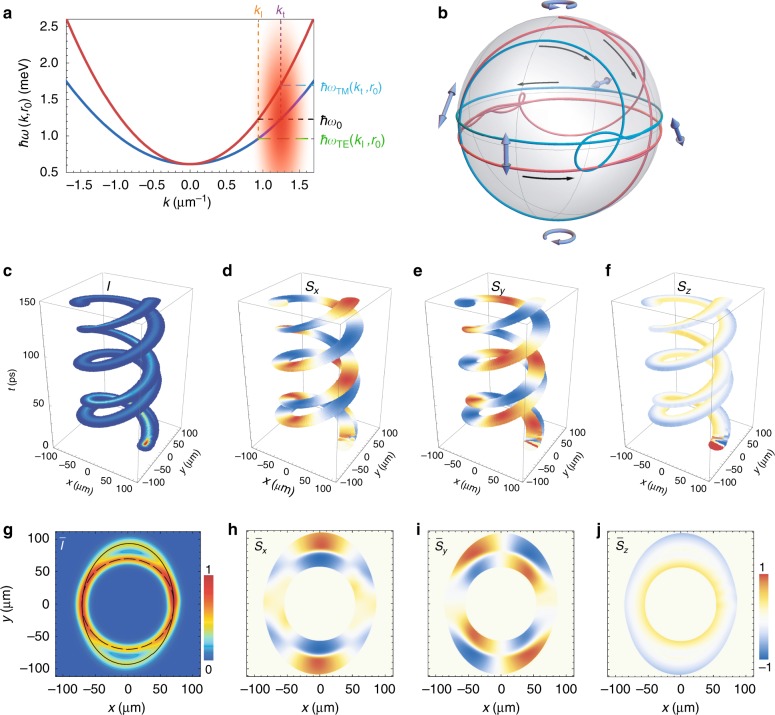
Polarization behaviour of a sub-picosecond polariton pulse. **a** Dispersion of two spin-split polariton modes $\hbar \omega _{{\text{TE}},{\text{TM}}}(k,r_0)$ at position *r*_0_ = 70 μm in comparison with the spectrum of the probe pulse. The ultrashort probe pulse of 200-fs duration is resonant to the TE polariton mode: *k*_p_ = *k*_t_ and *ω*_p_ = *ω*_0_ at *r*_0_. **b** Trajectories on the Poincaré sphere of the Stokes vector characterizing polarization of the two spatio-temporal components of the split sub-picosecond polariton pulse. The black arrows indicate the direction with respect to the evolution in time. **c** Evolution of the intensity and **d**–**f** polarization of the two components of the sub-picosecond polariton pulse. **g** Time-integrated spatial distribution of the normalized intensity and **h**–**j** polarization components of the polariton pulse. The polarization of the probe pulse is taken as right circular for (**c**)–(**j**). The dashed and solid closed black curves in panel (**g**) indicate the trajectories of the TE and TM components of the polariton pulse, respectively

The above calculations have been performed under the adiabatic limit, implying that the energy level separation, which is the TE-TM splitting $\Delta _{{\text{LT}}}k_{\text{p}}^2$, is large in comparison with the characteristic energy scales of the system, including the spectral width of the polariton pulse. Let us now consider the polarization dynamics of the sub-picosecond polariton pulse possessing an energy spectrum with a margin overlapping both the TE and TM dispersion branches. For definiteness, we take the pulse duration as *w*_T_ = 200 fs. The other characteristics of the probe pulse are the same as those in Fig. 2, i.e., *x*_p_ = *x*_0_, *ω*_p_ = *ω*_0_ and *k*_p_ = *k*_t_. The initial polarization is taken to be right circular as well. The excitation spectrum is schematically shown in Fig. 5a. In the considered excitation regime, the polariton wave packet predictably separates into two spatial components with orthogonal polarizations; see Fig. 5c–f. The time-integrated intensity and polarization components in Fig. 5g–j show clear separation of the trajectories of the wave packet components. The TE-branch component resonant to the probe excitation follows the circular inner trajectory of radius *r*_0_ (the black dashed ring in Fig. 5g), while the TM-branch component propagates along the elliptic outer trajectory with the small ellipse axis close to 2*r*_0_ (the black solid ellipse in Fig. 5g). The trajectories of the Stokes vectors characterizing polarization of the components of the polariton wave packet on the Poincaré sphere are shown in Fig. 5b. The conclusion made earlier is also valid beyond the adiabatic limit: the fraction of the circular polarization becomes negligibly small in both components of the polariton wave packet over the course of evolution. However, this fraction is noticeably smaller for the TE-branch component.

**Fig. 6:**
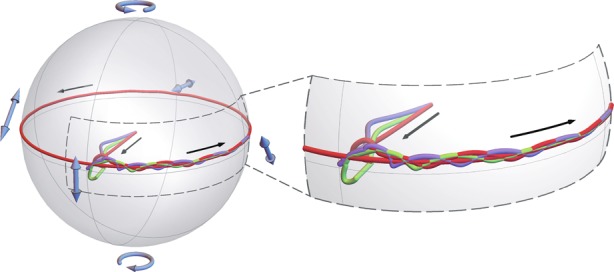
Trajectories on the Poincaré sphere of the Stokes vector characterizing the polarization of the polariton pulse excited by an unpolarized probe. The three curves represent the results of three numerical experiments, with the initial conditions representing a random distribution of polarization $| f \rangle$ of the probe pulse over space and time. The right inset shows an enlarged image of the selected area

The discussion above is related to the operation with polariton pulses excited by a polarized input. The remarkable peculiarity of the considered system is that a polariton pulse excited by an initially unpolarized probe acquires linear polarization in the course of propagation. The results of modelling confirming this statement are shown in Fig. 6. The trajectory of the Stokes vector on the Poincaré sphere degenerates to the closed one, analogous to the characteristic of pulses excited by a polarized probe (see Fig. 2b). Therefore, in addition to the allocated function of the polarization rectifier, the considered system is also able to act as a polarizer for polariton pulses. It is advantageous over the traditional polarizer due to the fact that full intensity of the probe is used to excite the polariton pulse, which implies reduced losses connected with reflection or absorption of the objectionable component of polarization.

## Discussion

In this paper, we report the concept of a spinoptronic device able to transform polariton pulses with a given polarization to linearly polarized pulses. The basis of the operating principle of the device is a separation of eigenmodes of the system with orthogonal linear polarizations both in real and reciprocal space due to the mutual impact of the confining potential and the TE-TM splitting. We have shown that the spatial separation in linear polarization is also valid for ultrashort (sub-picosecond) pulses beyond the adiabatic limit.

One should note here that the effect of the separation of the linear polarizations of light can be observed even in a pure photonic structure representing an empty optical microcavity modified by a confining potential for photons in the cavity plane. However, the proposed system based on the strong-coupling regime has two undeniable advantages in terms of applications over the pure photonic system.

In the pure photonic scheme with an empty microcavity, the lifetime of the optical pulse is fully determined by the quality factor of the cavity. In recent papers^33,34^, it was reported on ultra-high-Q-factor microcavities providing photon lifetimes of several hundreds of picoseconds. The scheme proposed in our manuscript allows an extremely long lifetime of propagating polariton pulses, which in theory can be infinitely large even in microcavities of relatively low quality. For this goal, we suggest using the effect of the stimulated scattering from the reservoir of incoherent excitons to the coherent polariton state. The reservoir excitons are created optically by the subthreshold spatially homogeneous non-resonant cw pump, which itself does not create the polariton condensate. When polaritons with the given quasimomentum *k* are injected with the resonant probe pulse, the density of polaritons with this *k* increases locally, which triggers the process of stimulated scattering to the *k*-state at the position of the pulse. The closer the non-resonant pump power is to the condensation threshold and the weaker the polariton pulse, the more effective is the feeding of the polariton pulse from the exciton reservoir. Remarkably, in the proposed scheme, the pulse lifetime is determined not by the polariton or photon lifetime but by the inflow from the exciton reservoir. In this regard, the pulse lifetime can exceed that of polaritons and photons by many orders, and achieving several nanoseconds for the lifetime of the polariton pulses seems to be a routine procedure.

Another advantage of the polariton-based device over the pure photonic device is the controllability of the former. Due to the sensitivity of the exciton component to an external impact (see, e.g. refs. ^35–38^), one can tune the system to choose the required operating frequency range, the TE-TM splitting energy, and the shape of the dispersion surface. Remarkably, the tuning can be performed at any stage of working with the system, both in the stage of structure growth and after the growth of the structure is complete.

The proposed scheme has application potential far beyond its use as a polarization rectifier. In particular, we have demonstrated that linear polarization is acquired by a pulse excited by an unpolarized pulsed input during its propagation. This allows the considered system to function as a polarizer for polariton pulses. The scheme can act as a laboratory tool for the study of polariton phenomena, including circular polarization supercurrents, interference effects and spin-orbit interaction effects of long-living macroscopic polariton states.

## Methods

We model the dynamics of the polariton pulses based on the generalized Pauli equation for the spinor $\left. {|{\mathrm{\Psi }}} \right\rangle = \left[ {{\mathrm{\Psi }}_ + (t,{\mathbf{r}}),{\mathrm{\Psi }}_ - (t,{\mathbf{r}})} \right]^{\text{T}}$, where ${\mathrm{\Psi }}_ \pm (t,{\mathbf{r}})$ are the wave functions of the right- and left-circularly polarized polariton components:

2 $$\left. {{\mathrm{i}}\hbar \partial _t|{\mathrm{\Psi }}} \right\rangle = \left[ {\hat H_0 + {\mathrm{i}}\hbar (Rn_{\text{R}} - \gamma _{\text{C}})/2} \right]\left. {|{\mathrm{\Psi }}} \right\rangle \left. {\,+\, {\mathrm{i}}|F(t,{\mathbf{r}})} \right\rangle$$

where, in addition to the conservative Hamiltonian $\hat H_0$ given in (1), we introduce non-conservative processes of pumping and loss, with *γ*_C_ being the polariton decay rate. The polaritons are excited by the resonant probe $\left. {|F(t,{\mathbf{r}})} \right\rangle = \left. {F_{\text{T}}(t)F_{\mathbf{r}}({\mathbf{r}})|f} \right\rangle$. To preserve the shape of the polariton pulse, we take the spatial component of the probe in the Gaussian form $F_{\mathbf{r}} \propto {\mathrm{exp}}[ - ({\mathbf{r}} - {\mathbf{r}}_{\text{p}})^2/2w^2]\,{\mathrm{exp}}({\mathrm{i}}{\mathbf{k}}_{\text{p}}{\mathbf{r}})$ of width $w = \sqrt {\hbar /m^ \ast \omega _{{\text{tr}}}}$. The pulse is shifted both in real and reciprocal space to **r**_p_ and **k**_p_, respectively; see Fig. 1 for clarity. The temporal component is $F_{\text{T}} \propto {\mathrm{exp}}[ - t^2/2w_{\text{T}}^2]\,{\mathrm{exp}}( - {\mathrm{i}}\omega _{\text{p}}t)$, where *w*_T_ is the duration of the pulse and *ω*_p_ is the probe frequency. The vector $\left. {|f} \right\rangle = \left[ {f_ + ,\,f_ - } \right]^{\text{T}}$ defines polarization of the probe. For better perception, in the model, we restrict ourselves by choosing the characteristics of the probe pulse, *r*_p_, *k*_p_ and *ω*_p_, such that the trajectory of the pulse is close to a circular one taking *r*_p_ = *r*_0_, *ω*_p_ = *ω*_0_ and *k*_p_ = *k*_l,t_.

To increase the pulse lifetime, we allow the reservoir of excitons to partially feed the polariton condensate; *n*_R_ is the density of the non-resonantly pumped reservoir of excitons, and *R* is the stimulated scattering rate describing the particle exchange between the reservoir and the pulse. We consider the reservoir to be pumped by a non-resonant homogeneous pump *P* slightly below the condensation threshold, $P \,<\, P_{{\text{th}}} = \gamma _{\text{C}}\gamma _{\text{R}}/R$, where *γ*_R_ is the exciton decay rate. For the low-intensity pump and probe, we assume that the reservoir density is homogeneous as well, $n_{\text{R}} \simeq P/\gamma _{\text{R}}$. For this reason, we do not include interaction effects characteristic of dense systems.

The values of the parameters used for modelling are as follows. The effective polariton mass is $m^ \ast = 7 \times 10^{ - 5}m_{\text e}$, where *m*_e_ is the free electron mass. The TE-TM splitting constant is $\hbar \Delta _{{\text{LT}}} = 300\,{{\upmu} \mathrm{eV}}\,{{\upmu} {\text{m}}}^2$. The polariton and exciton decay rates are $\gamma _{\mathrm{C}} = 0.02\,{\text{ps}}^{ - 1}$ and $\gamma _{\text{R}} = 0.025\,{\text{ps}}^{ - 1}$, respectively. The condensate-reservoir coupling rate is $\hbar R = 0.05\,{\text{meV}}\,{{\upmu} \text{m}}^2$. The non-resonant subthreshold homogeneous pump is taken as $P = 0.95P_{{\text{th}}}$. The harmonic trap frequency is taken as $\omega _{{\text{tr}}} = 25\,{\text{GHz}}$.
